# Evaluation of WHO Measles Eradication Programme for the European Region of 53 Countries with Emphasis on Poland in the Years 2003–2014

**DOI:** 10.3390/tropicalmed10020043

**Published:** 2025-02-05

**Authors:** Dominika Mucha, Beata Łubkowska, Joanna Jeżewska-Frąckowiak, Piotr M. Skowron

**Affiliations:** 1 Department of Health and Natural Sciences, Division of Biochemistry, Gdansk University of Physical Education and Sport, 80-308 Gdansk, Poland; beata.lubkowska@awf.gda.pl; 2 Department of Molecular Biotechnology, Faculty of Chemistry, University of Gdansk, 80-308 Gdansk, Poland; j.jezewska-frackowiak@ug.edu.pl (J.J.-F.); piotr.skowron@ug.edu.pl (P.M.S.)

**Keywords:** epidemiological surveillance for measles, infectious diseases, measles, vaccine, WHO measles eradication programme, epidemiology

## Abstract

Background: The vaccination programmes after the 2nd World War and the application of safe and effective vaccines were expected to eliminate infectious diseases within the World Health Organization (WHO) European Region. However, epidemiological indexes show isolated cases and local epidemiology outbreaks of viral measles, that draw attention to re-evaluate the effectiveness and obstacles of infectious disease eradication programmes. Methodology: This study analyses the available Polish governmental epidemiological data concerning the WHO European Region for the years 2003–2014 and evaluates the effectiveness of the WHO Measles Eradication Programme implementation, since 2001. Most of these epidemiological data are either available in Polish or scattered in governmental reports only. Thus, we have compiled selected available data to present an overview focusing on Poland’s measles epidemiological situation. Results: The analysis of the available data clearly shows that infection cases of measles are on the rise in the European Region or are steady at a relatively high level depending on the country. The critical factors to prevent measles are maintaining a vaccination level at a minimum of 95% using two doses of MMR, adequate infection detection, rapid reporting, controlling and enforcing identification, and mass media campaigns to inform societies about the necessity of measles vaccination and the safety of using MMR. Conclusions: Besides the current SARS-CoV-2 pandemics, measles is possibly the most dominating infectious disease on the rise in the European Region, including Poland. The eradication programme modifications to increase its effectiveness are of upmost importance, as measles is one of the most infectious diseases with acute syndromes, especially affecting children.

## 1. Introduction

The current SARS-CoV-2 pandemic has prompted societies and governments to realisations that epidemics are not a past history of humankind. This includes not only unprecedented events in modern times (after the Second World War), including coronavirus’s extremely rapid outbreak and high death toll, exceeding 4 mln people worldwide, but also points to a potential for novel pandemics, including those caused by pathogens thought to be either eliminated or completely marginalised. Historically, vaccine application in Europe dates back at least to the XVIII century; one of the more documented developments was the progress in smallpox vaccine production in 1796 by Edward Jenner [1]. Further, the vaccination concept has expanded to other human diseases as well as veterinary applications [2,3]. However, unexpectedly, despite tens of years of preventive vaccination programmes using new-generation effective vaccines in European countries, in the XXI century, on one hand, complete eradication of some infectious diseases has taken place, but on the other, many others are returning, with noted multiple infection cases or even local outbreaks. This includes one of the most infectious viral diseases—measles. This disease, mainly affecting children, results in long-term, dangerous and life-threatening complications. The infection is passed through contact with a person who exhibits measles symptoms. The etiological factor is the measles virus, which belongs to the *Morbilivirus* genus, family *Paramyxoviridae*, whose genetic material is composed of negatively polarised single-stranded RNA [4]. An acute variant of the disease is observed essentially among children, with typical symptoms such as high fever, rash, gingivitis, headache, and photophobia [5]. Thus far, the most effective preventive method against measles is active protection, i.e., vaccination.

Since 2001, the European Region has been subjected to the Measles Eradication Programme coordinated by WHO, whose major goal is taming virus transmission and finally achieving a complete eradication in all 53 countries of the region. Despite the current problems with increasing measles cases, the disease meets the criteria for complete eradication: humans are the only reservoir of the virus, neither symptomless infections nor carriage has been observed, the environment is not contaminated with the pathogen, and effective vaccines are available [4]. Nevertheless, social anti-vaccination movements, which have been active for the last several years, strongly promote avoiding vaccinations and have led to many children being unprotected from infectious diseases. This general public perception is linked to a publication from 1998 by Lancet, suggesting that MMR vaccines increase the probability of developing a non-specific, intestine inflammatory syndrome and autism in vaccinated children [6,7]. The publication was later withdrawn since it was based on unproven and non-representative data. Moreover, numerous further studies have shown that there is no such link; however, the matter remains controversial, especially in mass media and general public perception [6].

An additional factor apparently linked to measles cases and outbreaks in the European Region is the rapid increase in human migrations across the region, from various countries, both within and from outside of Europe, including global migrations, where preventive vaccinations are either not conducted, or they are not enforced by law. In this paper, we summarise and comment on epidemiological data in the years 2003–2014 in Poland concerning this increasingly troubling measles problem, some of which are difficult to access or in incomplete governmental reports only, and/or not available in English. We are currently continuing to collect data and preparing compilations concerning the next period, covering the years 2015–present. Due to limited data availability from local clinics and the substantial travelling effort needed for gathering representative data, here we publish those for 2003–2014. New data will be published as an update for 2015–2024.

## 2. Materials and Methods

Based on Polish and international publication data, the progress and effectiveness of the WHO Measles Eradication Programme in the European Region, including Poland, was evaluated for a period of nine years (2003–2014), and infection and outbreak cases were compared, as were additional, detailed factors linked to measles vaccinations.

### 2.1. Measles Definition

Measles is an acute and extremely infectious viral disease displaying numerous harsh syndromes, typically follicular-blemished rash, fever and at least one of the following: cough, nose rhinitis and ophthalmic [8]. The etiological factor is an RNA virus, classified as a *Morbilivirus* belonging to the *Paramyxoviridae* family. The only source of the measles virus is humans, with an infection route via droplets. Measles develops in four stages: incubation, prodrome periods, rash period, and recovery [9]. The incubation period is typically hard to detect, as it is mostly symptomless. During the heralds period high fever appears, up to 40 °C, along with ophthalmia, lacrimation, photophobia, catarrh of nasal mucous membranes, and dry cough. In the oral cavity, mucous membranes develop small white spots on a red background, known also as Fiłatow-Koplik’s spots, separated by a mucous membrane of natural colour. This stage typically lasts 1–3 days. Next comes the rash stage, usually 14 days from the initial exposure to the measles virus. The rash begins behind the ears and around the mouth, and then spreads to the chin, torso and limbs. Initially, the rash is lumpy and dark pink, later it changes to a brick-red colour, and it further spreads and fuses with other rashes, typically covering the majority of the skin surface, leaving tracts of white, unaffected skin. At the end of the 3rd or 4th day, the rash disappears in reverse order than it had developed, leaving greyish brown skin discolourations. The recovery period starts the moment the rash disappears; in addition, the fever decreases and the patient’s general state noticeably improves. On the skin of the face and torso minute, furfuraceous skin flakes develop. The most frequent complications include pneumonia, bronchitis, otitis media, and laryngitis subglottic. The risk of developing neurological complications increases with the age of a sick child, over the age of 9 it is 2–3-fold higher. These complications include measles-inflicted encephalitis, myelitis, Guillain-Barré syndrome, and subacute sclerotic leucoencephalitis, which cause permanent damage to motoric and mental capabilities. Gastrointestinal tract complications include stomach and intestine inflammation, liver inflammation, and appendix inflammation, among others [5,9,10].

### 2.2. Vaccination Schemes

As a result of the WHO Measles Eradication Programme for the European Region, introduced in the year 2001, until the year 2011, most of the region’s countries (51 out of 53) had introduced trivalent vaccines against measles, mumps, and rubella (MMR). The Russian Federation has introduced MMR vaccines in selected regions, while in Tajikistan only a monovalent vaccine against measles was introduced [11].

According to the scheme against measles adopted in Poland, vaccinations with the trivalent MMR vaccine are conducted twice, the first time in the 13–14th month of infant life and the second in the 10th year of life [12,13]. This type of vaccine has been used in Poland since 2004, whereas the first measles vaccinations in Poland were introduced in 1974. The former scheme included a single dose vaccination in the 2nd year of infant life using a monovalent preparation, containing an attenuated mixture of various strains of measles: Schwarz, Edmonston–Enders, Zagreb, and Leningrad [4,14]. Since 1991, two doses have been used. Routine vaccination against measles, mumps, and rubella (MMR) was introduced in Poland in 2004 [4]. Presently, the most often applicable MMR in Poland are M-M-RVAXPRO^®^ and PRIORIX^®^ which contain measles virus Enders–Edmonston (live, attenuated) produced in chick embryo cells, Mumps virus Jeryl Lynn strain (live, attenuated) produced in chick embryo cells, and Rubella virus Wistar RA 27/3 strain (live, attenuated) produced in WI-38 human diploid lung fibroblasts [4,15]. The vaccination against measles in Poland is obligatory.

### 2.3. WHO Measles Eradication Programme

In 2001, the Measles Eradication Programme coordinated by WHO, concerning all 53 countries of the European Region, including Poland, was introduced. The term ‘eradication’ with regard to measles is defined as follows: there are no endemic outbreaks and, in the case of virus transfer from unprotected regions, further virus transmission is not supported [16,17]. Critical conditions leading to measles eradication are (i) maintaining 95% of children vaccinated with the two doses of MMR within a given population, which results in group immunity; (ii) estimation of the remaining fraction of measles-sensitive persons within a population, as based on serological assays, detecting anti-measles IgMs, conducted on persons exhibiting measles-like symptoms; and (iii) the constant monitoring of the epidemiological situation by registering all the actual and suspected measles infection cases, followed by their laboratory confirmation or rejection by the WHO Reference Laboratory. In Poland, this function is conducted by the Department of Virology at the National Institute of Public Health—National Institute of Hygiene (NIPH-NIH). The measles eradication strategy assumes that the number of suspected measles cases would be 1 or more per 100,000 inhabitants. This translates to a minimum of 383 cases in Poland. Another assumption is to intensify the vaccination programme within national minorities, where frequently suspected measles cases are not reported to doctors and thus, infection case statistics are often underrepresented in these groups. Because measles is highly infectious, approaching 90% upon contact of a healthy person with an infected one, reporting suspected cases is obligatory in all 53 countries of the European Region [18].

### 2.4. Data Indentation, Extraction and Analysis

The data on measles cases in Poland were sourced from the annual articles “Odra w Polsce” (“Measles in Poland”) published in the “Przegląd Epidemiologiczny” (“Epidemiological Review”) and submitted to the Department of Epidemiology of Infectious Diseases and Surveillance at the National Institute of Public Health NIH-NRI by Provincial Sanitary and Epidemiological Stations as a part of the Statistical Research Program of Public Statistics, namely the following:

(1) Annual reports on infectious diseases, infections, and poisonings (governmental form MZ-56);
(2) Annual reports on selected infectious diseases by age, gender, place of residence, and seasonality (governmental form MZ-57);
(3) Annual reports on selected infectious diseases by vaccination status, gender, age, and place of residence (governmental form MZ-58);
(4) Periodic reports on cases and suspected cases of influenza (governmental form MZ-55) for the given year.

If data from other information sources were used in a given study, this is stated in the respective cited articles [16,17,19,20,21,22,23,24,25,26,27,28,29].

## 3. Results and Discussion

Based on the available literature and unpublished data, compilations showing statistics concerning measles for the European Region in the years 2003–2014 have been made, with emphasis on Poland. Data are presented in the form of graphs and tables and are further commented on.

### 3.1. Poland: Measles Reporting Criteria

Reporting measles within 24 h from diagnosis is a legal obligation in Poland according to the Legal Act from 5 December 2008 concerning the prevention and elimination of infections and infectious diseases [19]. However, statistics show that the period between a doctor’s inspection and reporting a suspected measles case to a local sanitary—epidemiological unit is substantially longer and takes on average 5 days [16]. The definitions of infectious disease cases for implemented procedures of the NIPH-NIH epidemiological supervision in Poland for measles are as follows [20]: (i) clinical criteria state every person exhibiting simultaneously fever and maculo-papular rash, as well as at least one of the following symptoms: cough, mucous catarrh of the nose, and conjunctivitis; (ii) laboratory criteria based on clinical samples isolated from a potentially infected person, include at least one of the following: isolation of the measles virus, detection of measles coding nucleic acid, or detection of anti-measles IgMs, specific for acute measles infection or detection of measles viral antigens in the serum or saliva, with the use of specific monoclonal antibodies during a direct immunofluorescent assay against measles. An important factor while interpreting laboratory tests includes the history of measles vaccinations. In the case of a recent vaccination, the measles antigen typing or genotyping of the virus is needed to confirm or reject the presence of wild-type measles [17]. Epidemiological criteria are defined as human-to-human infection transfer with several case definition variants: (a) case possible—every person meeting clinical criteria, (b) case probable—every person meeting both clinical and epidemiological criteria, and (c) case confirmed—every person who has not recently been vaccinated against measles and meets both clinical and laboratory criteria. In the case of recently vaccinated persons—everyone who turned out positive in tests for the presence of wild-type measles virus [20].

### 3.2. Poland: Historical Perspective and Measles Genotype Variants Landscape

Universal measles vaccination was introduced in Poland in 1975. Initially, a single dose of the measles vaccine, named Attenuvax^®^, was administered at 13–15 months of age. Starting in 1991, two doses of the single measles vaccine (Rouvax^®^) were given—one at 13–15 months of age and the second at 8 years of age. Since 2004, two doses of the combined MMR vaccine, protecting against measles, mumps, and rubella, have been administered. Initially, the MMR II vaccine was used. Currently, two formulations are available on the market: M-M-RvaxPro^®^ (since 2008, previously marketed as MMR II) and Priorix^®^ (available since 2002). The vaccines are administered in two doses: at 13–14 months of age and at 10 years of age. The composition of these vaccines differs slightly in the strains of attenuated live measles viruses they contain: (i) Priorix^®^—live attenuated measles virus (Schwarz strain) at a minimum dose of 10^3^ CCID50 and (ii) M-M-RvaxPro^®^—live attenuated measles virus (Enders-Edmonston strain) at a minimum dose of 1 × 10^3^ CCID50. Both vaccines contain the same quantity of viral particles but differ in the strain of the measles virus used. The decrease in measles cases can be attributed to the new regulations implemented in 2004, particularly the use of the new vaccines. However, the subsequent rise in measles incidence in later years is likely linked to the growing anti-vaccination movement and the increasing percentage of unvaccinated children. An analysis of data on all mandatory vaccinations in Poland reveals a significant increase in parental refusals over the years. In 2011, approximately 4700 children were not vaccinated due to parental refusal. This number rose to about 5300 in 2012, over 7000 in 2013, and exceeded 12,000 in 2014.

**Figure 1:**
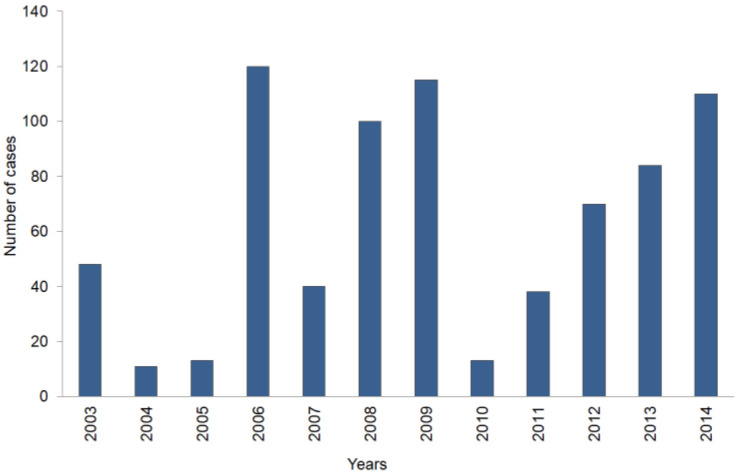
Compilation of confirmed measles infections in Poland during the years 2003–2014 (Figures S1 and S2).

**Figure 2:**
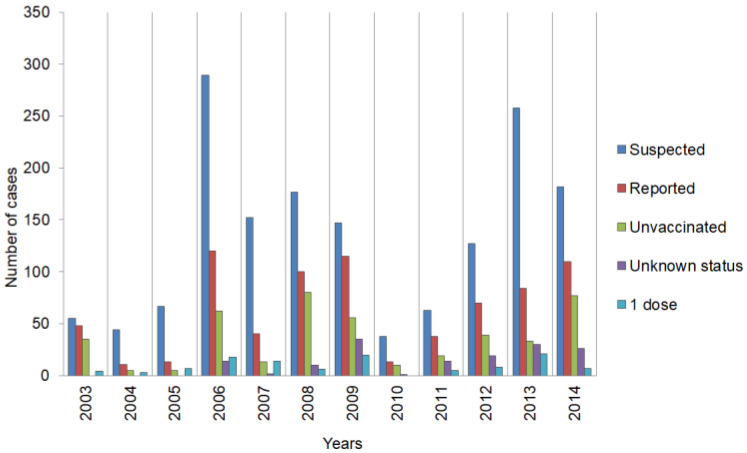
Compilation of total measles-related disease cases with symptoms, which are or can be connected with measles infection incidences in Poland during the years 2003–2014. The graph shows suspected and confirmed measles cases shown in relation to vaccination characteristics (Figures S1 and S2).

In Poland before the introduction of vaccination against measles, the virus infected up to 200,000 people yearly [4]. As a result of introducing the vaccination, both before and during the WHO Measles Eradication Programme implementation, the epidemiological status has greatly improved, by a factor of over 1000-fold, and statistical data are depicted in Table 1 and Table 2 and in Figure 1 and Figure 2. The data presented in Table 1 and Table 2 and Figure 1 and Figure 2 have been compiled from a series of publications [16,17,21,22,23,24,25,26,27,28,29,30,31,32,33,34].

Data provided by the Central Statistical Office (GUS) indicate that between 2003 and 2014, Poland’s population changed only slightly, increasing from 38.19 million in 2003 to 38.478 million in 2014, with an average population of 38.303 million during this period. To ensure better comparability and account for changes in health policies, the results were standardised according to the European standard population [31]. Due to the lack of data divided into age groups, detailed standardised calculations were not possible. However, calculations were performed considering population size and event rates per 100,000 individuals. Event rates per 100,000 individuals show significant differences between years. For example, the lowest event rate was recorded in 2004 (0.029 per 100,000), while the highest was observed in 2006 (0.315 per 100,000). Between 2010 and 2012, a decline was evident, followed by an increase in 2013 and 2014.

They evidently show the highest sensitivity of detection of various syndromes, which might be related to measles, was obtained in 2006. This is related to an increased preventive activity of the Polish healthcare system, induced by a measles epidemic in Ukraine, where by the end of 2006 the number of registered and confirmed measles cases, inflicted by measles virus genotype variant D6, reached 30,000 [35,36]. In genetic studies of measles viruses isolated in Warsaw, small local outbreaks were caused by two genotype variants—D4 and D5, which are not related to the ones that caused the Ukraine epidemic. However, three persons residing in Ukraine during the D6 epidemic outbreak had developed measles symptoms after returning to Poland. This is an indication of a more general trend in the countries in the European Region, which have a complex genotype pattern, due to frequent reintroduced by importation and spread through highly mobile, unvaccinated groups, frequently of common ethnic origin [27,37,38,39]. However, it is also important to note, that the measles virus possesses stable antigenic architecture and all its strains belong to the same serotype. Thus, those closely related strains’ differentiation is based on genetic differences. Thus far, 8 strain groups were differentiated (A–H), which include 23 genotypes [35,36]. Sequence analysis of the 3′ nucleoprotein gene, coding for the C-terminus of the protein has been established as a marker region for the classification of clinical isolates as a particular measles strain. Some of the genotypes (B1, E, F, G1, D1) are denoted as a ‘non-active’ state, which is defined as a lack of isolation of a given strain for 15 years. All measles virus strains used for vaccine production generally belong to genotype A. However, in Europe strains belonging to genotypes D4, D5, and D6 have been observed in major part [35,36]. Genotype D6 was associated with the Ukraine outbreak, while genotype D4 was detected in an outbreak in Romania. These two strains are highly related. In addition, the decreased diversity of the genotype D6 is detected as well as the rapid disappearance of the previously circulating C2 and D7 viruses. These observations lead to the conclusion that vaccination programmes had worked well and succeeded in the interruption of chains of transmission. However, at the same time, a concurrent process was taking place—continuous reintroduction by importation and spread via the highly mobile European Region population, with measles viruses being continually imported from external sources. This results in a wobbling genotype landscape due to the number of susceptible individuals increasing and the transmission of the newly introduced viral genotypes. So on one hand, eradication programmes result in overall virus quenching, manifested infected cases with viruses with very limited genetic diversity and on the other hand possible rapid changes in the endemic genotypes [27,37,38,39,40]. Nevertheless, it is important to point out that no meaningful biological differences were detected among measles viruses of various genotypes—neither in clinical symptoms nor in post-infection complications. Thus, genetic differentiation is an important tool for the monitoring of outbreaks and virus transmissions as well as for molecular epidemiology studies [35,36]. Thus, measles virus regional tracking is possible, exemplified by studies of transfer between Ukraine and Poland. Even though both countries share long boundaries and substantial travelling occurring between these countries, only a very limited increase in measles cases in Poland occurred—120 confirmed infections as compared to 13 in 2005, which indicates the presence of a very effective measles control system operating in Poland [28]. Nevertheless, the following years showed that the control system was not ideal, as there was a relatively low number of suspected cases, which indicates an insufficient system of reporting all suspected symptoms. Since the maximum of measles infections in 2006, the number of observed cases was gradually declining until 2010. Then, a constant growth of reported cases was observed until 2014, reaching a relatively high level, similar to the one in 2006 [20,30].

### 3.3. Poland: Social and Local Populations Factors

Since there were no measles epidemics in any nearby countries, it is highly probable that other factors had become dominant, such as anti-vaccination social movements. Even though the paper from 1998 by Lancet [7] was dismissed and numerous detailed studies, including those in Poland, show no connection between MMR vaccinations and autism, as well as other complications, the turmoil caused by this publication is still deeply rooted in societies [6]. This has caused the vaccination rate to decrease as a result of a number of parents’ decisions not to vaccinate children, the phenomenon is also clearly observed in Poland. As an apparent consequence, measles infections and local outbreaks are steadily increasing [18,30,40]. It has been shown that measles cases are underreported in areas occupied by ethnic minorities such as the Romani people and there are more frequently observed local outbreaks in their communities. Thus, it is desirable to increase the control system’s effectiveness, especially in territories populated by minorities [16]. After analysing the degree of vaccinations among children and young people in Poland, it is evident that it is very high, with the first MMR dose (Table 3) [20]; however, the following years show a decline in the vaccination level. The dominant fraction of infected people comprises those who have not been vaccinated, for whom the vaccination status is unknown or who have received a single MMR dose only.

### 3.4. Other European Region Countries

**Figure 3:**
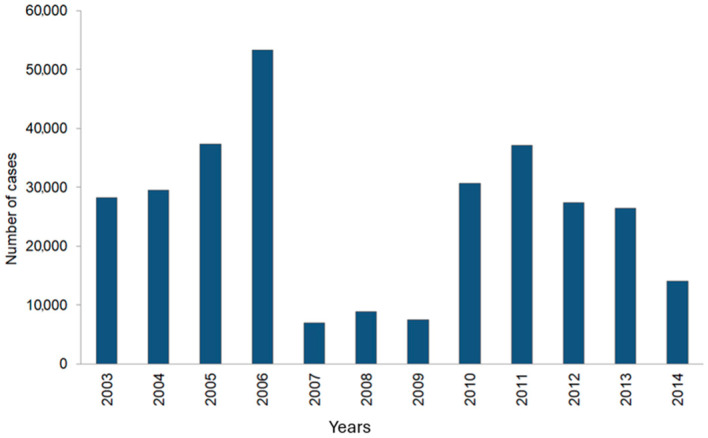
Reported measles cases in the European Region during the years 2003–2014 Figure S3.

**Figure 4:**
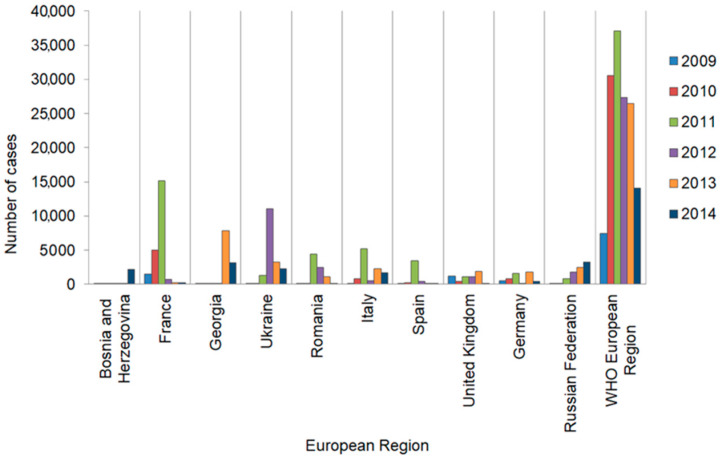
European Region countries with the highest numbers of measles cases reported during the years 2003–2014 Figure S3.

Statistical data derived from foreign publications and WHO reports for 53 countries of the region are analysed below. These include: (i) Western European Sub region: Andorra, Austria, Belgium, Cyprus, Denmark, Finland, France, Germany, Greece, Island, Ireland, Israel, Italy, Luxemburg, Malta, Monaco, Holland, Norway, Portugal, San Marino, Spain, Sweden, Switzerland, and United Kingdom; (ii) Central and Eastern European Sub region: Albania, Bosnia-Herzegovina, Bulgaria, Croatia, Czechs, Estonia, Hungary, Lithuania, Latvia, Montenegro, Poland, Romania, Serbia, Slovakia, Slovenia, Macedonia, and Turkey; (iii) Sub region of ex-Soviet Union Countries: Armenia, Azerbaijan, Belarus, Georgia, Kazakhstan, Russian Federation, Ukraine, Moldovia, Kirgizstan, Tajikistan, Uzbekistan, and Turkmenistan [41]. Measles and measles-related cases are shown in Table 3 and Table 4 and Figure 3 and Figure 4 based on the compiled data from the literature [11,30,41,42,43,44,45].

Measles is a disease which is required to be strictly reported in all 53 countries of the European Region and the data obtained in each country is submitted in the form of yearly reports to the WHO database. All these countries are encouraged to prepare and submit to WHO additional information, such as the age of infected people, their vaccination status, laboratory confirmations of measles cases and detected viral genotypes, among others. Despite continued inquiries, currently, only a limited number of the European Region countries send such extended monthly reports [11]. According to the WHO Programme regulations, since 2001 efforts have been made to maintain a high percentage of population vaccination, reaching in many countries of the region as much as 95% [11]. After analysing available data, it is evident that during the years 2007–2009, a noticeable decline of measles cases in the European Region was observed, after ceasing the Ukrainian epidemic. Nevertheless, since 2010, the number of measles cases has been on the rise. The highest number of reported cases was noted in France and Spain. It was determined that this was mainly due to local outbreaks within ethnic groups of immigrants who had limited access to national healthcare programmes. Among them were those whose religious or other beliefs prevented them from undergoing vaccinations and people afraid of post-vaccination complications [41]. This trend, which started in 2010, is still observed, with reported measles cases systematically on the rise or stabilised on a relatively high level, as compared to previous years. Somewhat surprisingly and increasingly problematic is the fact that this negative scenario is linked to people refusing vaccinations in the most highly developed countries. This is in part a result of anti-vaccination social movements, which lead to lowering the number of vaccinated children and thus generating permissive targets for the virus. This phenomenon primarily concerns developed European countries, where a liberal approach to vaccinations is most pronounced. Even though Poland is not yet in this group, the same negative scenario is observed: for example, the vaccination rate indicating the first MMR dose in 2012 was 99.6%, in 2013—99.5%, and in 2014—98.5% [46]. Although a high vaccination percentage of 95% is reported, the trend shows a progressing decline. Overall, the situation is complicated by recent numerous wars outside the European Region, with the NATO army involved. Due to the expected high exposure to various infectious diseases, including measles, and inherently compromised sanitary standards during military operations, it is certainly difficult, if not locally impossible, to maintain conditions recommended by WHO and imposed by the Measles Eradication Programme. Another consequence of military operations is that vaccination programmes, as well as measles reporting systems, are operating partially or not operating at all in affected areas. Further complications, especially evident since 2015, include the uncontrolled migration of hundreds of thousands to millions of people from war-affected areas in Northern Africa, the Middle East and Ukraine to the European Region, which results in an inflow of a population with a high percentage of formally unknown vaccination status, but in reality—unvaccinated.

## 4. Conclusions

1. Despite the long history of measles vaccination programmes aiming at the elimination of epidemics and eradication of the measles virus, the analysis of the available data clearly shows that infection cases are on the rise in the European Region or are steady at a relatively high level, depending on the country.
2. Regardless of the reason, the decrease in the percentage of vaccinations leads to the formation of a measles-sensitive fraction of the population, not limited to children, but concerning all age groups.
3. The evident rise in measles infections in developed Western European countries, leading to local outbreaks, is troubling.
4. The necessary condition for measles eradication is strict adherence to the WHO Measles Eradication Programme for the European Region, which aims at infection eradication during 2012–2020 [47].
5. Besides maintaining a vaccination level at a minimum of 95% using two doses of MMR, the critical factors seem to be adequate infection detection, rapid reporting, controlling and enforcing identification, and mass media campaigns to inform societies about the necessity of measles vaccination and safety of using MMR.

## 5. Paper Context

The vaccinations after the Second World War were expected to eliminate infectious diseases. However, epidemiological indexes show local epidemiology outbreaks of measles. Data concerning Poland were scattered in governmental reports in Polish only. The analysis shows that infection cases of measles are on the rise in Poland and in other parts of the European Region. The eradication programme modifications are needed, as measles is one of the most infectious diseases with acute syndromes, especially affecting children.

## Figures and Tables

**Table 1:** Measles cases in Poland during the years 2003–2014. The number of infections and death cases as well as infections and death cases per 100,000 people (Figures S1 and S2).

Year	2003	2004	2005	2006	2007	2008	2009	2010	2011	2012	2013	2014
The number of measles cases in Poland	48	11	13	120	40	100	115	13	38	70	84	110
Population size	38,190	38,173	38,157	38,125	38,115	38,135	38,167	38,529	38,538	38,533	38,495	38,478
Number of cases per 100,000 people	0.126	0.029	0.034	0.315	0.105	0.262	0.301	0.034	0.099	0.182	0.218	0.286

**Table 2:** Suspected and confirmed measles cases in Poland during the years 2003–2014, profiled to vaccination characteristics and schemes, vaccinated child’s age, and nationality.

Year		2003	2004	2005	2006	2007	2008	2009	2010	2011	2012	2013	2014
Status measles cases in Poland	Suspected	55	44	67	289	152	177	147	38	63	127	258	182
	Reported	48	11	13	120	40	100	115	13	38	70	84	110
	Unvaccinated	35	5	5	62	13	80	56	10	19	39	33	77
	Unknown	0	0	0	14	2	10	35	1	14	19	30	26
	1 dose	4	3	7	18	14	6	20	0	5	8	21	7

**Table 3:** Reported measles cases in the European Region during the years 2003–2014.

Year	2003	2004	2005	2006	2007	2008	2009	2010	2011	2012	2013	2014
The number of measles cases in WHO European Region	28,203	29,510	37,338	53,344	6936	8879	7499	30,625	37,101	37,101	26,436	14,059

**Table 4:** European Region countries with the highest numbers of measles cases reported during the years 2003–2014 Figure S3.

Year		2009	2010	2011	2012	2013	2014	Total Number of Measles Cases in Countries 2009–2014
**Countries in the WHO European Region**	Bosnia and Herzegovina	22	45	10	22	5	2204	2308
	France	1541	5019	15,214	754	272	267	23,067
	Georgia	23	22	64	31	7868	3190	11,198
	Ukraine	24	42	1313	11,086	3308	2326	18,099
	Romania	8	187	4417	2447	1159	53	8271
	Italy	173	861	5179	530	2251	1676	10,670
	Spain	43	285	3507	401	131	153	4520
	United Kingdom	1176	397	1083	1093	1900	137	5786
	Germany	572	805	1600	139	1781	436	5333
	Russian Federation	101	152	783	1771	2500	3248	8555
	WHO European Region	7499	30,625	37,101	27,379	26,436	14,059	143,099

## Data Availability

The original contributions presented in this study are included in the article/Supplementary Materials. Further inquiries can be directed to the corresponding author(s).

## Supplementary Materials

The following supporting information can be downloaded at: https://www.mdpi.com/article/10.3390/tropicalmed10020043/s1, Figure S1: Compilation of confirmed measles infections in Poland during the years 2003–2014; Figure S2. incidences in Poland during the years 2003–2014. The graph shows suspected and confirmed measles cases shown in relation to vaccination characteristics.; Figure S3. Reported measles cases in the European Region during the years 2003–2014.

## References

[1] Barquet N., Domingo P. (1997). Smallpox: The Triumph over the Most Terrible of the Ministers of Death. Ann. Intern. Med..

[2] Kumaran S., Deivasigamani B., Alagappan K.M., Sakthivel M. (2010). Infection and immunization trials of Asian seabass (*Lates calcarifer*) against fish pathogen *Vibrio anguillarum*. J. Environ.Biol..

[3] Ha Y.M., Kim Y.I., Kim K.H., Kim S.K. (2008). Neutralization of white spot syndrome virus (WSSV) for Penaeus chinensis by antiserum raised against recombinant VP19. J. Environ. Biol..

[4] Makówka A., Gut W., Litwińska B. (2007). Podstawy programu eliminacji odry na świecie i w Polsce [Measles elimination programme on the world and Poland]. Przegląd Epidemiologiczny [Epidemiol. Rev.]..

[5] Dziubek Z. (2010). Choroby Zakaźne i Pasożytnicze [Infectious and Parasitic Diseases].

[6] Mrozek-Budzyń D., Kiełtyka A., Majewska R. (2009). Brak związku między szczepieniami skojarzoną szczepionką przeciw odrze, śwince i różyczce (MMR) a występowaniem autyzmu u dzieci—Wynik badania kliniczno-kontrolnego [Lack of association between MMR vaccination and the incidence of autism in children: A case-control study]. Przegląd Epidemiologiczny [Epidemiol. Rev.].

[7] Dyer C. (2010). Lancet retracts Wakefield’s MMR paper. Br. Med. J..

[8] Magdzik W., Naruszewicz-Lesiuk D., Zieliński A. (2007). Choroby Zakaźne i Pasożytnicze—Epidemiologia i Profilaktyka [Infectious and Parasitic Diseases—Epidemiology and Prevention].

[9] Cianciara J., Juszczyk J. (2012). Choroby Zakaźne i Pasożytnicze [Infectious and Parasitic Diseases].

[10] Magdzik W., Naruszewicz-Lesiuk D. (2001). Zakażenia i Zarażenia Człowieka. Epidemiologia, Zapobieganie i Zwalczanie [Human Infections and Infestations. Epidemiology, Prevention and Control].

[11] Centers for Disease Control and Prevention (CDC) (2009). Progress toward measles elimination--European Region, 2005–2008. MMWR Morb. Mortal. Wkly. Rep..

[12] Ministry of Health, Republic of Poland Act of 18 August 2011 on Mandatory Vaccinations, 2011. https://isap.sejm.gov.pl/isap.nsf/DocDetails.xsp?id=WDU20111821086.

[13] (2012). Communicate of Chief Sanitary Inspectorate of the State Sanitary Inspection (SSI) of 29th October 2012 r. on Mandatory Vaccinations for 2013, 2012, Dz.U.. https://dziennikmz.mz.gov.pl/DUM_MZ/2012/78/akt.pdf.

[14] Janaszek W., Gut W., Gay N.J. (2000). The epidemiology of measles in Poland: Prevalence of measles virus antibodies in the population. Epidemiol Infect..

[15] Freestone D.S., Prydie J., Smith S.G., Laurence G. (1971). Vaccination of adults with Wistar RA 27/3 rubella vaccine. J. Hyg..

[16] Karasek E., Paradowska-Stankiewicz I. (2013). Measles in Poland in 2011. Przegl. Epidemiol..

[17] Karasek E., Rogalska J., Paradowska-Stankiewicz I. (2012). Odra w Polsce w 2010 roku [Measles in Poland in 2010]. Przegl. Epidemiol..

[18] Nickerson E., Simon K. (2008). Choroby Zakaźne.

[19] Ministry of Health, Republic of Poland Act of 5th December 2008 on the Prevention and Control of Infections and Infectious diseases in Humans. 2008, Dz.U. 2016, 1866. https://isap.sejm.gov.pl/isap.nsf/DocDetails.xsp?id=WDU20160001866.

[20] National Institute of Hygiene (PZH) (2014). Definicje Przypadków Chorób Zakaźnych na Potrzeby Nadzoru Epidemiologicznego. (Case Definitions of Infectious Diseases for Epidemiological Surveillance Purposes). http://wwwold.pzh.gov.pl/oldpage/epimeld/inne/Def_PL2_3.pdf.

[21] Rogalska J. (2011). Odra w Polsce w 2009 roku [Measles in Poland in 2009]. Przegl. Epidemiol..

[22] Rogalska J. (2010). Odra w Polsce w 2008 roku [Measles in Poland in 2008]. Przegl. Epidemiol..

[23] Stefanoff P., Rogalska J. (2009). Odra w Polsce w 2007 roku [Measles in Poland in 2007]. Przegl. Epidemiol..

[24] Stefanoff P., Czarkowski M.P., Kondej B. (2007). Odra w 2005 roku [Measles in 2005]. Przegl. Epidemiol..

[25] Stefanoff P., Czarkowski M.P., Kondej B. (2006). Odra w 2004 roku [Measles in 2004]. Przegl. Epidemiol..

[26] Stefanoff P., Czarkowski M.P. (2005). Odra w 2003 roku [Measles in Poland in 2003]. Przegl. Epidemiol..

[27] Santibanez S., Tischer A., Heider A., Siedler A., Hengel H. (2002). Rapid replacement of endemic measles virus genotypes. J. Gen. Virol..

[28] Stefanoff P., Czarkowski M.P., Kondej B. (2008). Odra w 2006 roku [Measles in 2006]. Przegl. Epidemiol..

[29] Rogalska J., Karasek E., Paradowska-Stankiewicz I. (2014). Odra w Polsce w 2012 roku [Measles in Poland in 2012]. Przegl. Epidemiol..

[30] World Health Organization (WHO) (2015). Measles Reported Cases by Country. http://apps.who.int/gho/data/view.main.1540_62?lang=en.

[31] Satman I., Bayirlioglu S., Okumus F., Erturk N., Yemenici M., Cinemre S., Gulfidan G., Arga K.Y. (2023). Estimates and forecasts on the burden of prediabetes and diabetes in adult and elderly population in Turkiy. Eur. J. Epidemiol..

[32] How Has the Measles Vaccine Been Used in the Past?. https://szczepienia.pzh.gov.pl/faq/jak-szczepionka-przeciw-odrze-byla-stosowana-w-przeszlosci/.

[33] Historical Immunization Programs. https://szczepienia.pzh.gov.pl/kalendarz-szczepien/.

[34] Population Structure by Age in the Years 1970–2060. https://stat.gov.pl/obszary-tematyczne/ludnosc/ludnosc/struktura-ludnosci,16,1.html.

[35] Częścik A., Trzcińska A., Siennicka J. (2011). Wirus odry—Reakcje odpornościowe związane z naturalnym zakażeniem oraz odpowiedzią poszczepienną [The measles virus—Immunologic reactions connected with natural infection and vaccination response]. Adv. Microbiol..

[36] Rota P.A., Featherstone D.A., Bellini W.J. (2009). Molecular epidemiology of measles virus. Curr. Top. Microbiol..

[37] Kremer J.R., Brown K.E., Jin L., Santibanez S., Shulga S.V., Aboudy Y., Demchyshyna I.V., Djemileva S., Echevarria J.E., Featherstone D.F. (2008). High genetic diversity of measles virus, World Health Organization European Region, 2005–2006. Emerg. Infect. Dis..

[38] Mulders M.N., Nebie Y.K., Fack F., Kapitanyuk T., Sanou O., Valéa D.C., Muyembe-Tamfum J.J., Ammerlaan W., Muller C.P. (2003). Limited diversity of measles field isolates after a national immunization day in Burkina Faso: Progress from endemic to epidemic transmission?. J. Infect. Dis..

[39] Rima B.K., Earle J.A., Yeo R.P., Herlihy L., Baczko K., Ter Meulen V., Carabana J., Caballero M., Celma M.L., Fernandez-Munoz R. (1995). Temporal and geographical distribution of measles virus genotypes. J. Gen. Virol..

[40] Rogalska J. (2015). Measles in Poland in 2013. Przegl. Epidemiol..

[41] Centers for Disease Control and Prevention (CDC) (2011). Increased Transmission and Outbreaks of Measles—European Region 2011. MMWR Morb. Mortal. Wkly. Rep..

[42] World Health Organization (WHO) (2016). Reported Measles Cases and Incidence Rates by WHO Member States 2013, 2014 as of 11 February 2015. https://immunizationdata.who.int/global/wiise-detail-page/measles-reported-cases-and-incidence?CODE=Global&YEAR=.

[43] Martin R., Wassilak S., Emiroglu N., Uzicanin A., Deshesvoi S., Jankovic D., Goel A., Khetsuriani N. (2011). What Will It Take to Achieve Measles Elimination in the World Health Organization European Region: Progress From 2003–2009 and Essential Accelerated Actions. J. Infect. Dis..

[44] World Health Organization (WHO) Status Report on Progress Towards Measles and Rubella Elimination SAGE Working Group on Measles and Rubella (22 October 2012). https://terrance.who.int/mediacentre/data/sage/SAGE_Docs_Ppt_Nov2012/7_session_measles-rubella/Nov2012_session7_progress_measles-rubella_elimination.pdf.

[45] O’Connor P., Jankovic D., Muscat M., Ben-Mamou M., Reef S., Papania M., Singh S., Kaloumenos T., Butler R., Datta S. (2017). Measles and rubella elimination in the WHO Region for Europe: Progress and challenges. Clin. Microbiol. Infect..

[46] The National Institute of Public Health—National Institute of Hygiene in Poland (NIPH–NIH) (2016). “Vaccinations in Poland” (Annual Report). http://wwwold.pzh.gov.pl/oldpage/epimeld/index_a.html#05.

[47] WHO Global Measles and Rubella Strategic Plan 2012–2020. https://iris.who.int/bitstream/handle/10665/44855/9789241503396_eng.pdf.

