# Supplementary figures and images for: Evaluation of WHO Measles Eradication Programme for the European Region of 53 Countries with Emphasis on Poland in the Years 2003–2014
Source: Trop Med Infect Dis. 2025 Feb 5;10(2):43. doi: 10.3390/tropicalmed10020043 (PMC11860599; doi:10.3390/tropicalmed10020043)

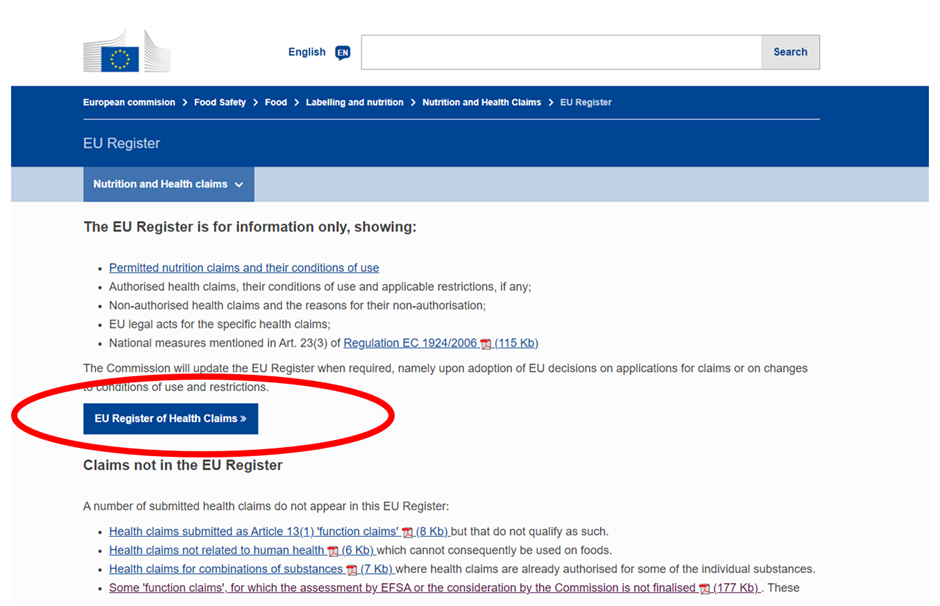

Supplement: Supplementary file 1 [file tropicalmed-10-00043-s001.zip › Figure S1.tif]

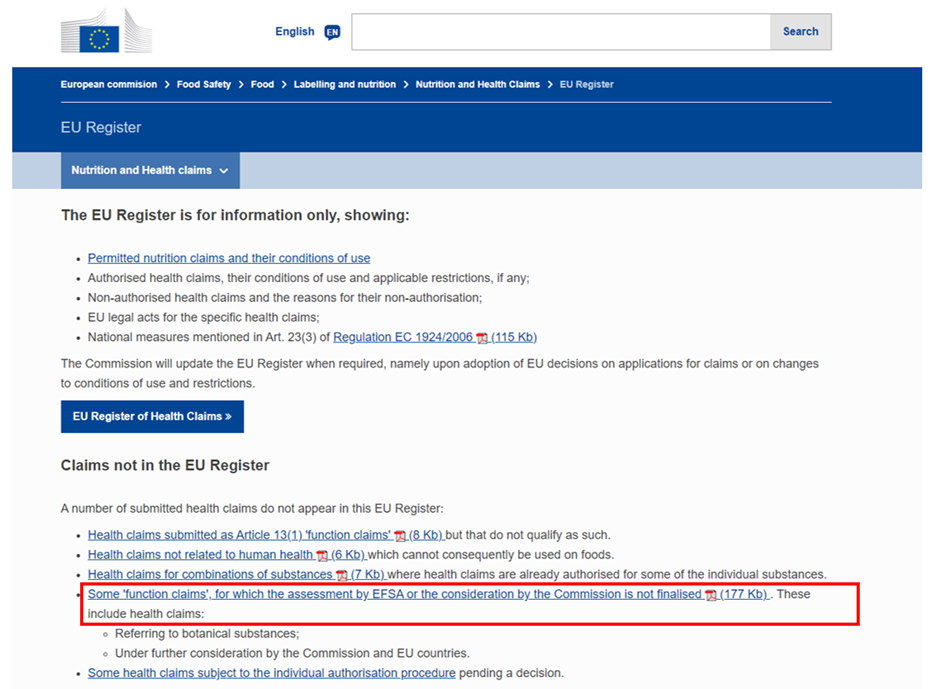

Supplement: Supplementary file 1 [file tropicalmed-10-00043-s001.zip › Figure S2.tif]

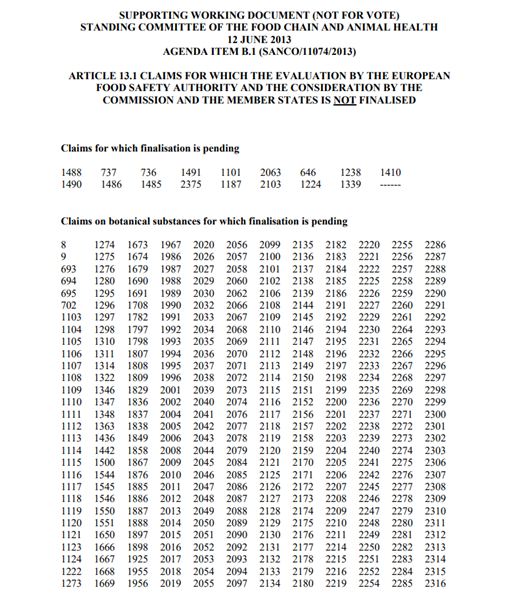

Supplement: Supplementary file 1 [file tropicalmed-10-00043-s001.zip › Figure S3.tif]
